# Using Subjective and Objective Measures of Financial Situation to Improve Program Outcomes for Older Adults

**DOI:** 10.1093/geroni/igaa057.2598

**Published:** 2020-12-16

**Authors:** Hector Ortiz, Jan Mutchler

## Abstract

Researchers in the field of aging rely on various measures of financial security to assess the needs of older adults and the outcomes of interventions. Recently, subjective measures have gained attention among researchers and organizations that serve older adults. This symposium brings together researchers from academia, government, and the non-profit sector to discuss the relationship between subjective financial well-being and objective financial situation. The first project describes the relationship between the Consumer Financial Protection Bureau’s Financial Well-being Score and the Elder Index. The CFPB Financial Well-Being Score provides a standardized and validated measure of a person’s sense of financial security and freedom in the present and future. The Elder Index provides a measure of older adults’ income against the average income needed for adults age 65 or older to live independently in their communities. The second project discusses the findings of a study into the changes in outcomes among older adults assisted through the National Council on Aging’s Benefits Enrollments Centers. The third project describes the overall findings and changes in financial well-being among SCSEP participants who attended a series of financial education workshops offered through the Benjamin Rose Institute on Aging’s subsidiary Empowering and Strengthening Ohio’s People. Together, the studies show that both measures are, on average, strongly correlated and predictive of a range of factual experiences such as material hardship and financial stress. The studies, however, show that subjective measures may help identify and target underlying behavioral and attitudinal factors that influence people’s satisfaction with their economic situation.

